# Egg-Albumin and Glucose Test Meal

**Published:** 1911-10

**Authors:** 


### ABSTRACTS

### Egg-Albumin and Glucose Test Meal

DR. MAURICE LOEPER, of Paris, *Le Progres Medical,* August 26, 1911, advocates a test meal consisting of two hundred grams of 30 per cent. mixture of coagulated egg-albumin with 10 per cent. of glucose. This has a volume of 250 c.c. and contains exactly eight grams of albumin, twenty of glucose and two hundred and ten of water. Its molecular concentration is 0.83. Even by extracting with the aid of wash water, titration of the sugar readily allows an estimate of the amount of the test meal remaining in the stomach. The stomach contents are evacuated at the end of twenty-five minutes, when the total quantity should be about 80 c.c., and the molecular concentration reduced to 0.60. The rapidity of the latter change corresponds to the gastric circulation, being slow in cases of weak heart and low arterial tension and vice versa. Various tests, some quite analogous to those commonly practised, others of a more recondite nature are made. In Ulcer, 1 gram of albumin remains; in hypochlohydria, 7 grams; in cancer 6 grams; in cancer with pyloric stenosis the same—in typic cases.

